# Microcystin-LR-Exposure-Induced Kidney Damage by Inhibiting MKK6-Mediated Mitophagy in Mice

**DOI:** 10.3390/toxins15060404

**Published:** 2023-06-19

**Authors:** Xueqiong Yao, Ying Liu, Yue Yang, Yafang Li, Na Hu, Fengmei Song, Fei Yang

**Affiliations:** 1 Department of Epidemiology and Health Statistics, The Key Laboratory of Typical Environmental Pollution and Health Hazards of Hunan Province, School of Basic Medicine, School of Public Health, Hengyang Medical School, University of South China, Hengyang 421001, China; 2 Hunan Provincial Key Laboratory of Clinical Epidemiology, Department of Social Medicine and Health Management, Xiangya School of Public Health, Central South University, Changsha 410017, China; 3 Key Laboratory of Environmental Medicine Engineering, Ministry of Education, School of Public Health, Southeast University, Nanjing 210000, China

**Keywords:** microcystin-LR, kidney damage, MKK6, mitophagy

## Abstract

Previous studies have reported that microcystin-LR (MC-LR) levels are highly correlated with abnormal renal function indicators, suggesting that MC-LR is an independent risk factor for kidney damage. However, the evidence for the exact regulation mechanism of MC-LR on kidney damage is still limited, and further in-depth exploration is needed. In addition, the mitochondria-related mechanism of MC-LR leading to kidney damage has not been elucidated. To this end, the present study aimed to further explore the mechanism of mitophagy related to kidney damage induced by MC-LR through in vitro and in vivo experiments. Male C57BL/6 mice were fed with a standard rodent pellet and exposed daily to MC-LR (20 μg/kg·bw) via intraperitoneal injections for 7 days. Moreover, HEK 293 cells were treated with MC-LR (20 μM) for 24 h. The histopathological results exhibited kidney damage after MC-LR exposure, characterized by structurally damaged nephrotomies, with inflammatory cell infiltration. Similarly, a significant increase in renal interstitial fibrosis was observed in the kidneys of MC-LR-treated mice compared with those of the control group (CT) mice. MC-LR exposure caused impaired kidney function, with markedly increased blood urea nitrogen (BUN), creatinine (Cr), and uric acid (UA) levels in mice. Ultrastructural analysis exhibited obviously swollen, broken, and disappearing mitochondrial crests, and partial mitochondrial vacuoles in the MC-LR-treated HEK 293 cells. The Western blotting results demonstrated that exposure to MC-LR significantly increased the protein expressions of MKK6, p-p38, and p62, while the expression of mitophagy-related proteins was significantly inhibited in the kidneys of mice and HEK293 cells, including parkin, TOM20, and LC3-II, indicating the inhibition of mitophagy. Therefore, our data suggest that the inhibition of MKK6-mediated mitophagy might be the toxicological mechanism of kidney toxicity in mice with acute exposure to MC-LR.

## 1. Introduction

In recent years, global cyanobacteria blooms have become increasingly frequent with the intensification of eutrophication and global warming trends. Cyanobacteria and cyanobacteria toxin pollution has become a major environmental problem faced by China and even the world for a long time [1,2,3]. A worldwide survey showed the detection of microcystins (MCs) in parts of Europe, North America, Australia, Asia, and Africa, particularly in tropical and subtropical regions [4]. Microcystin-LR (MC-LR) is a widely distributed cyanobacteria toxin, characterized by hepatotoxicity, and has multi-organ toxicities such as cardiovascular toxicity, reproductive toxicity, neurotoxicity, and genotoxicity [5,6,7,8,9]. MC-LR can affect human health via a variety of ways, including via contaminated food, water, air, physical contact with contaminated items, and hemodialysis [5], and the International Agency for Research on Cancer classifies it as a Group 2B carcinogen [10]. The World Health Organization (WHO) and the National Health Commission of China stipulate that the content of MC-LR in drinking water for adults and children should not exceed 1 μg/L [11].

In addition to the liver, the kidneys are also one of the major target organs for the specific distribution of MC-LR [12,13]. The kidneys function in the metabolism and excretion of drugs and poisons. Their blood flow is abundant, accounting for 25% of cardiac output, and when nephrotoxic substances are present in the blood, they can be highly vulnerable [13,14]. Godd et al. have shown that MC-LR enters the digestive tract through drinking or eating MC-LR-contaminated water or food and is transported and absorbed in the gastrointestinal tract, and then some of that MC-LR accumulates in the organs, and some of that MC-LR enters the blood, is transported to all parts of the body with the blood, and, finally, is excreted through the kidneys [15]. The transport mechanism of renal cells is similar to that of hepatocytes, and excess MC-LR can be transported into renal cells via specific organic anion transport polypeptides (OATPs) [16,17], causing damage to the structure and function of kidneys in fish and mammals [18,19,20,21,22].

Growing epidemiological evidence has suggested that MC-LR could cause kidney damage. Studies have pointed out that MC-LR levels are highly correlated with abnormal renal function indicators, suggesting that MC-LR might be an independent risk factor for renal impairment [23]. In addition, our previous case-control study based on a large-scale population survey also found that MC-LR was an independent risk factor for chronic kidney disease (CKD) [24]. However, there is limited evidence for the regulation mechanism of MC-LR on kidney damage, and further in-depth exploration is needed.

A study with 3 different doses (25, 75, and 100 μg/kg body weight) of MC-LR treatment in grass carp suggested that MC-LR could lead to the structural destruction of the kidneys, and massive swollen mitochondrial and vacuolar degeneration was found using transmission electron microscopy [25]. In addition, apoptosis features such as chromatin concentration and cell contraction were observed in the high-dose infected group [26]. Mitogen-activated protein kinase kinase 6 (MKK6) is an upstream kinase of p38 mitogen-activated protein shock (p38MAPK) that directly activates p38MAPK and features in multiple signal transduction pathways induced by environmental stress. Activating the p38MAPK signaling pathway leads to the reorganization of hepatocytes and the neuroendocrine cytoskeletal structure and the hyperphosphorylation of cytoskeleton-associated proteins. However, the role of MC-LR in the kidneys via the MKK6/p38 pathway has not been reported [27].

Mitochondrial dysfunction is involved in the occurrence and progression of many pathological processes. The selective elimination of damaged and depolarized mitochondria is called mitophagy. Mitophagy plays a critical role in maintaining mitochondria’s normal function [28,29]. In recent years, studies have shown that abnormal mitophagy could lead to acute kidney injury, and targeted intervention in mitophagy might improve the prognosis of kidney injury [30]. Mitophagy requires the coordination of the two processes of activating autophagy and initiating mitochondria. There are two main mitochondrial initiation mechanisms, and the PTEN-induced putative kinase 1 (PINK1)/E3 ubiquitin ligase Parkin (PARK2)-mediated pathway carries more weight [31,32]. Tubular epithelial cells are rich in mitochondria, supporting kidney function. When a kidney is damaged, the tubular epithelial cells are the staple site of cell damage and death. Fragmented or swollen mitochondria, stromal vacuole formation, mitochondrial ridge disappearance, and other damaged manifestations exist. When mitophagy is defective, damaged mitochondria not only cause cellular energy metabolism disorders but are also a major source of reactive oxygen species (ROS) [29,33]. Mitophagy reduces mitochondrial ROS and mitochondrial-DNA-mediated inflammatory responses by removing damaged mitochondria, reducing mitochondrial-ROS-mediated oxidative stress, and inhibiting subsequent apoptosis [29,34,35]. Furthermore, studies have reported that p38 MAPK inhibitors can promote PINK1-Parkin-mediated mitophagy by inhibiting Parkin serine 131 phosphorylation [34,35,36].

However, the mitochondria-related mechanism of MC-LR mediated via the MKK6/p38 signaling pathway leading to kidney damage has not been elucidated. The present study aimed to further explore the mechanism of MKK6/p38-mediated mitophagy related to kidney damage induced by MC-LR through in vitro and in vivo experiments.

## 2. Results 

### 2.1. Characteristics of MC-LR-Exposed Mice

**Figure 1:**
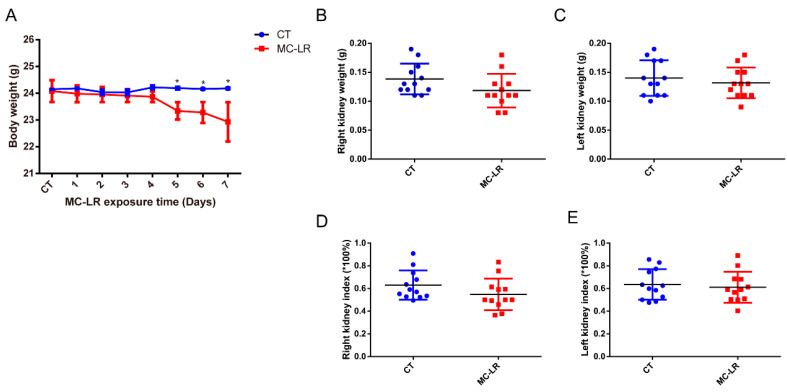
Characteristics of MC-LR-exposed mice. (**A**) Body weight curve of MC-LR exposed mice; (**B**) right kidney weight; (**C**) left kidney weight; (**D**) right kidney index; (**E**) left kidney index. Data are presented as means ± SD (*n* = 12). CT, normal control; MC-LR, microcystin-leucine-arginine-exposed. Symbols: * *p* < 0.05 vs. CT group.

After 7 days of feeding, no death or symptoms in the MC-LR mice treated daily with a dose of 20 μg/kg·bw of MC-LR were observed during the experiment, and all the mice were generally in good condition. As shown in Figure 1A, during the whole experimental period, the body weights of mice in the control (CT) group showed a rising trend, while those in the MC-LR group showed the reverse. Moreover, after 5 days of treatment with MC-LR, the body weights of the MC-LR mice were significantly lower than those of the CT mice, and this difference continued until the end of the experiment (Figure 1A; *p* < 0.05). However, there was no significant difference in kidney weight between the MC-LR group and the CT group (Figure 1B,C; *p* > 0.05). In addition, the kidney indices were also not altered after exposure to MC-LR, with no significance in the kidney indices between the 2 groups (Figure 1D,E; *p* > 0.05). 

### 2.2. Effects of MC-LR on Parameters Related to Kidney Function in Mice

**Figure 2:**
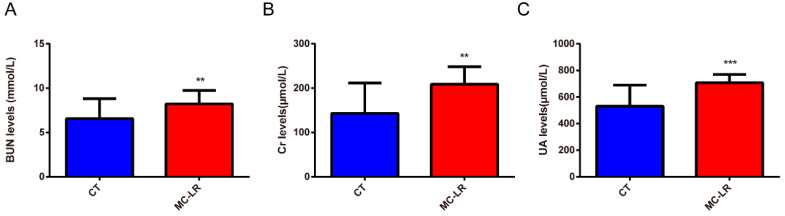
Kidney-function-related circulatory parameters. (**A**) BUN levels in MC-LR-exposed mice; (**B**) Cr levels in MC-LR-exposed mice; (**C**) UA levels in MC-LR-exposed mice. BUN, blood urea nitrogen; Cr, serum creatinine; UA, uric acid. Data are presented as means ± SD (*n* = 12). Symbols: ** *p* < 0.01, and *** *p* < 0.001 vs. CT group.

Next, we examined kidney-function-related parameter levels in the mice serum using colorimetry. As shown in the Figure 2 below, compared with the CT group, the serum blood urea nitrogen (BUN) levels were markedly increased after exposure to MC-LR for 7 days (Figure 2A; *p* < 0.05). In addition, the MC-LR group manifested significantly higher levels of creatinine (Cr) and uric acid (UA) (Figure 2B,C; *p* < 0.05). All these results suggest that MC-LR induced kidney function damage. 

### 2.3. Histopathology in Kidney

**Figure 3:**
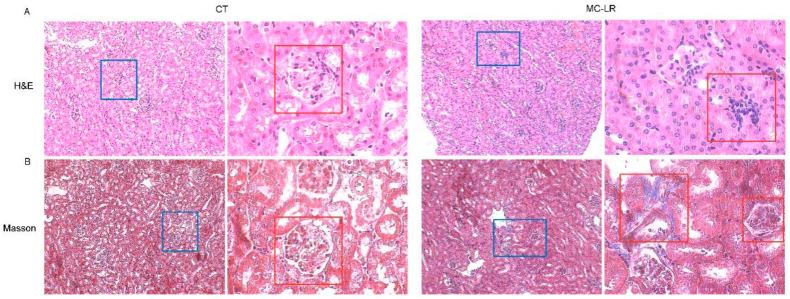
Histopathological morphological images. (**A**) Representative HE staining of the kidneys of MC-LR-exposed mice (magnification 100×, 400×; the blue and red boxes show the representative nephrosomes); (**B**) representative Masson staining of mice kidneys (magnification 100×, 400×; the blue and red boxes show the representative nephrosomes).

Histological analysis showed destroyed nephrosomes with fatty infiltration in the MC-LR mice in comparison with the CT mice, with significant tubular dilatation and atrophy and interstitial inflammation in the kidneys of the MC-LR mice (Figure 3A). Similarly, the renal fibrosis, assessed via Masson staining, showed significantly increased interstitial fibrosis in the kidneys of the MC-LR mice compared with those of the CT mice (Figure 3B).

### 2.4. Effect of MC-LR on Mitophagy-Related Protein Expressions in the Kidneys of Mice 

**Figure 4:**
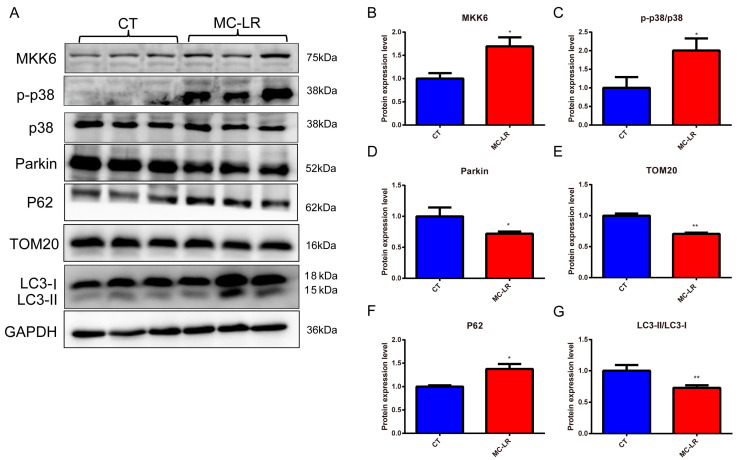
Effect of MC-LR on mitophagy-related protein expressions in the kidneys of mice. (**A**) Representative bands of Western blotting; (**B**) the expression of MKK6 normalized against GAPDH; (**C**) levels of phosphorylated p38 (p-p38) normalized against total p38; (**D**) levels of parkin normalized against GAPDH; (**E**) levels of p62 normalized against GAPDH; (**F**) levels of TOM20 normalized against GAPDH; (**G**) levels of LC3-II/LC3-I. Data are presented as means ± SD (*n* = 3). Symbols: * *p* < 0.05, and ** *p* < 0.01 vs. CT group.

In order to investigate whether mitophagy was activated in the kidneys of the MC-LR mice, we first detected the mitophagy-related protein expressions in the kidneys of the mice. As shown in Figure 4, the expression protein levels of MKK6, p-p38, the mitophagy protein parkin, the receptor protein p62, the mitochondria-associated protein TOM20, and the autophagy marker protein LC3-II were measured in total protein extracts from the kidneys. The results demonstrated that MC-LR induced significant upregulation of the MKK6, p-p38, and p62 proteins (Figure 4B,C,F; *p* < 0.05), while the expressions of the proteins parkin, TOM20, and LC3-II were inhibited (Figure 4D,E,G; *p* < 0.05), indicating the inhibition of mitophagy.

### 2.5. Viability of HEK293 Cells Exposed to MC-LR

**Figure 5:**
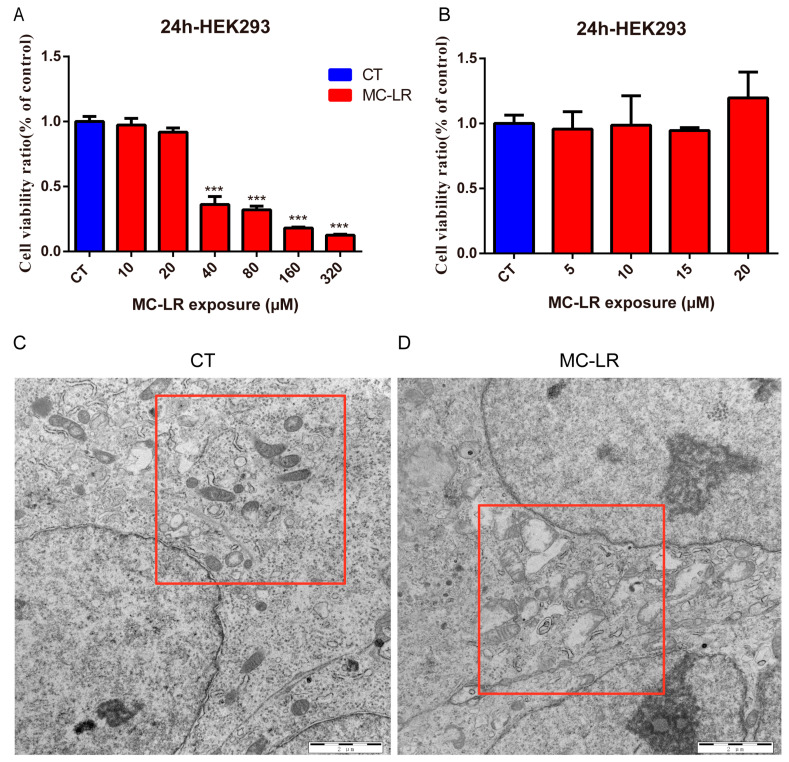
Effect of MC-LR on cell viability of HEK 293 cells and transmission electron microscopy. (**A**) Different concentrations of MC-LR treatment effects on cell viability of HER 293 cells. (**B**) HEK293 cell viability was determined with the CCK8 after exposure to 0–20 µM of MC-LR for 24 h. (**C**,**D**) Transmission electron microscopy images of CT and 20 µM MC-LR-treated HEK 293 cell ultrastructure (magnification 15,000×; red boxes show the representative mitochondria). Data are presented as means ± SEM (*n* = 3). Symbols: *** *p* < 0.001 vs. CT group.

HEK293 cells were exposed to different concentrations of MC-LR for 24 h, and cell viability was measured with the Cell Counting Kit-8 (CCK8). As shown in Figure 5A, MC-LR treatment inhibited cell viability in a dose-dependent manner. The IC_50_ value of MC-LR was 44 μM. Then, the HEK 293 cells were treated with different concentrations of MC-LR (0–20 μM) within 1/2 of the IC_50_ of MC-LR, and no effects were observed in the CCK8 assay after 24 h of MC-LR treatment (Figure 5B). Therefore, we used 20 μM (almost 1/2 of the IC_50_) of MC-LR for subsequent studies.

### 2.6. Effect of MC-LR on Mitophagy-Related Protein Expressions in HEK293 Cells

**Figure 6:**
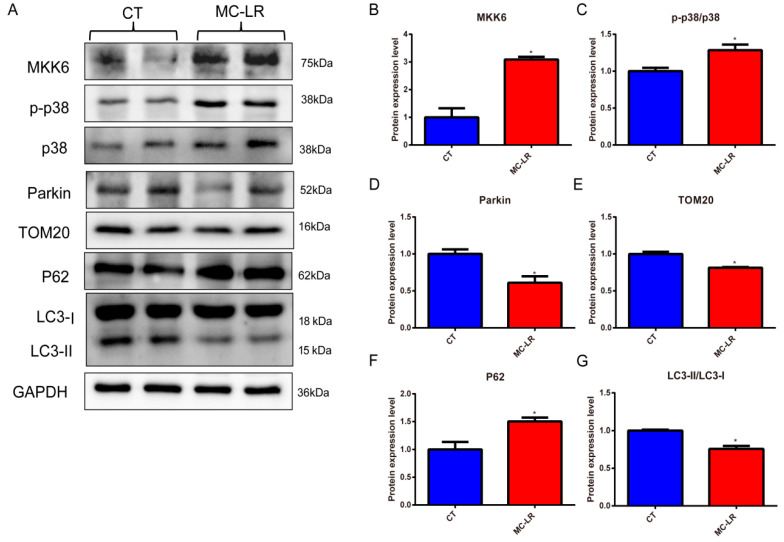
Effect of 20 µM (almost 1/2 of IC_50_) of MC-LR on mitophagy-related protein expressions in the HEK 293 cells. (**A**) Representative bands of Western blotting; (**B**) the expression of MKK6 normalized against GAPDH; (**C**) levels of phosphorylated p38 (p-p38) normalized against total p38; (**D**) levels of parkin normalized against GAPDH; (**E**) levels of p62 normalized against GAPDH; (**F**) levels of TOM20 normalized against GAPDH; (**G**) levels of LC3-II/LC3-I. Data are presented as means ± SEM (*n* = 3). Symbols: * *p* < 0.05 vs. CT group.

To investigate whether mitophagy was related to the pathogenesis of kidney damage, we detected morphological alterations in the mitochondria using transmission electron microscopy in the MC-LR-treated HEK293 cells. As shown in Figure 5D, we found obviously swollen, broken, and disappearing mitochondrial crests, and partial mitochondrial vacuoles (Figure 5C). In agreement with this, the expressions of MKK6, p-p38, and p62 dramatically increased in the MC-LR-treated HEK293 cells (Figure 6B,C,F; *p* < 0.05), while the expressions of mitophagy-related proteins, including parkin, TOM20, and LC3-II, were dramatically decreased (Figure 6D,E,G; *p* < 0.05), suggesting that MC-LR inhibited mitophagy in the HEK293 cells. These results corroborate the finding that MC-LR aggregated renal function injury in vivo.

## 3. Discussion

The maximum safe concentration of MC-LR in drinking water is set to 1 μg/L by the WHO [11]. In fact, the concentrations of MC-LR are far beyond that guideline in some waters. The concentration of microcystins even reached 15.6 μg/L in Lake Taihu, China [37], which could accumulate in aquatic creatures, and, thus, it poses a huge hazard to human health through the food chain. Moreover, another potential exposure to MC-LR in real life might be via the consumption of contaminated water. In addition, previous studies have reported that the LD50 value of MC-LR via the intraperitoneal route in mice was between 40–60 μg/kg·bw [38,39,40]. Therefore, we investigated the mechanism of MC-LR-induced kidney damage via intraperitoneal injection at a dose of 20 μg/kg·bw (almost 1/2 of the LD_50_) in the present study. 

Previous studies on the toxicity of MC-LR have focused on the liver and colon [19,41]. Recently, a study highlighted that MC-LR might play an important role in the pathological development and progression of kidney damage [18]. However, related investigations were only found in population studies. To our knowledge, this is the first study that has investigated the MKK6-mediated mitophagy involved in MC-LR-induced kidney damage in mice. Kidney tissue pathological lesions were observed in mice after 7 days of acute exposure to MC-LR. In addition, the ultrastructural analysis showed obviously swollen and broken mitochondria in MC-LR-treated HEK 293 cells as well as disappeared mitochondrial crests, and some mitochondria were vacuolized. Furthermore, the expression of the proteins MKK6 and p38, and the markers of mitophagy, including p62, TOM20, parkin, and LC3-II, were dramatically altered, indicating that MKK6-mediated mitophagy might be one of the mechanisms involved in MC-LR-induced kidney tissue damage. 

Consistent with previous observations [24,42], there was a significant difference in the body weights between the MC-LR mice and the CT mice, but no difference in kidney weights and kidney indices was found between the two groups. As for the physiological renal parameter, the results obtained indicate that exposure to MC-LR led to impaired renal function, and the serum Cr, BUN, and UA levels in the mice in the MC-LR group were significantly higher than those in the CT group, which is similar to the results found in the kidneys of zebrafish [42]. Additionally, the histological changes in the kidneys of the MC-LR mice once again confirmed the presence of kidney damage, with the destruction of the nephrosome structures and fibrosis in the renal tissues, which is consistent with previous findings [43,44,45,46]. 

Menezes et al. showed that exposure to high doses of MC-LR could lead to lysosomal instability and mitochondrial dysfunction in kidney cell lines, and autophagy was the early response of cells to toxins [26]. It has been suggested that MC-LR could induce the production of reactive oxygen species (ROS), subsequently triggering apoptosis in the kidneys of zebrafish [42]. Moreover, MC-LR exposure in the context of a high-fat/high-cholesterol/diet might increase the risk of developing renal casts [47]. In addition, the toxic effect of MC-LR manifested a time-dose dependence [48]. In summary, combined with previous studies, we know that exposure to MC-LR negatively affects the kidneys through pathological lesions. However, the possible mechanism of MC-LR causing nephrotoxicity demands further research.

Mitochondria are essential organelles for homeostasis, function, and viability in eukaryotic cells, which are the central hub of various signaling pathways for cell survival and death. Accordingly, maintaining mitochondrial homeostasis by balancing mitochondrial biogenesis and clearing damaged mitochondria is a key determinant of cell function [33]. It had been reported that mitochondria serve as the central mediators of MC-LR cytotoxicity, initiating different signaling pathways [49]. In the present study, the ultrastructural analysis observed swollen and broken mitochondria in MC-LR-treated HEK293 cells, along with partial autophagosomes and increased lysosomes, which is in parallel with the results of He’s group [25].

Mitogen-activated protein kinase kinase 6 (MAP2K6/MKK6), a bispecific protein kinase that plays an important role in regulating cellular responses to cytokines and various stresses, is an important component of the MAPK signaling pathway, especially in multiple signaling pathways induced by environmental stress [10,50]. MKK6 is the upstream kinase of p38MAPK, which can directly induce its activation, leading to transcription factor activation and gene expression regulation, resulting in kidney damage [27]. The downregulation of renal MKK6 and p-p38 expressions has a protective effect on diabetic kidney damage [51]. A previous study reported that an increased expression of MKK6, the activation of p38MAPK, and the ablation of p38α-coding genes can further enhance the formation of renal cysts and lead to renal cell carcinoma [52]. Furthermore, researchers found that the toxic effect of MC-LR on cells was closely related to the p38 signaling pathway. However, there was insufficient evidence for the mechanism by which MC-LR led to kidney damage via the MKK6/p38 MAPK signaling pathway.

**Figure 7:**
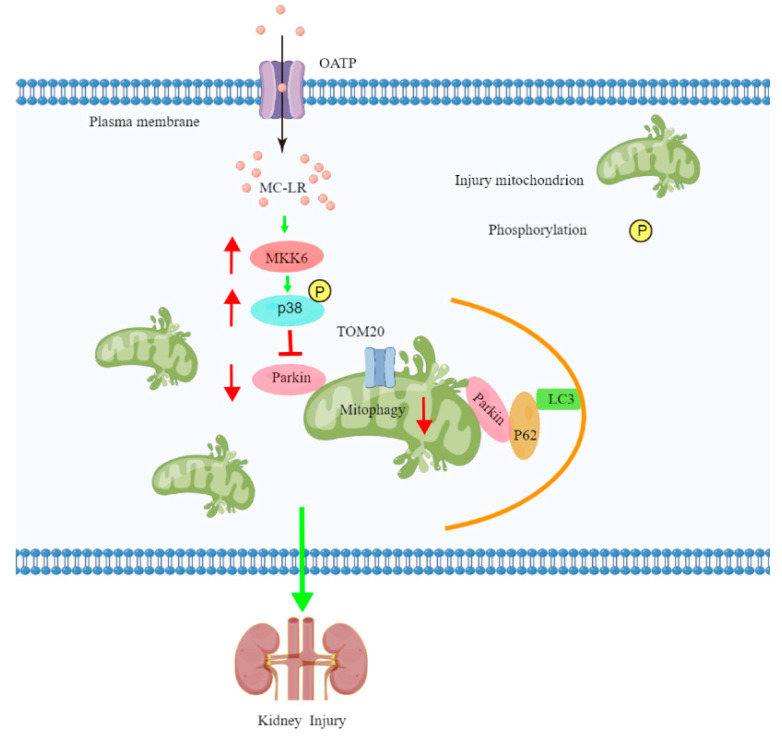
Molecular mechanism of MC-LR-exposure-induced kidney damage.

To further explore the mechanism of MC-LR-induced kidney damage, the expressions of the proteins MKK6 and p38, as well as the markers of mitophagy, including p62, TOM20, Parkin, and LC3-II, in the kidneys of mice and HEK 293 cells were determined using Western blotting. It has been shown that mitophagy is largely related to the E3 ubiquitin ligase parkin, which, in turn, induces mitophagy via regulating the mitophagy-related proteins p62, TOM20, and LC3-II [36,53,54]. Data from the present study indicate that exposure to MC-LR gave rise to the up-regulation of the expressions of MKK6, p-p38, p62, TOM20, and LC3-II and the down-regulation of the E3 ubiquitin-protein ligase parkin both in the kidneys of mice and HEK293 cells, which extends the previous studies asserting that mitophagy is a key cellular homeostatic mechanism activated early in acute kidney injury [28,33,53,55,56,57]. Consequently, our results suggest that MC-LR inhibited mitophagy via the MKK6/p38-dependent pathway, which is summarized in Figure 7.

## 4. Conclusions

Herein, we first reported that the inhibition of MKK6-mediated mitophagy was a significant contributor to kidney dysfunction and might be the toxicological mechanism of kidney toxicity in mice with acute exposure to MC-LR, a cyanobacterial heptapeptide toxin. And the mechanisms of chronic toxicity might be different.

## 5. Materials and Methods

### 5.1. Animals and Experimental Design

MC-LR was purchased from Alexis Corporation (Lausen, Switzerland), with a purity of more than 95%. C57BL/6 J mice were purchased from the Hunan SJA Laboratory Animal Co., Ltd. (Changsha, China). All procedures were approved by the Ethics Committee of the University of South China (XYGW-2018-41). A total of 24 healthy male mice at 6 weeks of age were randomly assigned to 2 groups—a control (CT) group and a microcystin-LR (MC-LR) group—and the MC-LR mice were intraperitoneally injected with MC-LR daily at a dose of 20 μg/kg·bw (approximately 1/2 of the LD_50_), for 7 days, continuously, which was based on the previously reported literature [38,39,40]. Meanwhile, the CT group was given a corresponding dose of saline solutions without MC-LR. The body weights of the mice were measured daily, and the entire experimental period was 7 days. The animal conditions were consistent with our previously published study [41].

### 5.2. Renal Function Indicators 

The blood samples were collected from the eyeballs of the mice, and the serum was separated via centrifugation at 4000× *g* for 30 min. The serum samples were deposited into clean 200 μL EP tubes, and the levels of the renal function indicators blood urea nitrogen (BUN), creatinine (Cr), and uric acid (UA) were determined using the BUN kit, Cr kit, and UA kit (Nanjing Jiancheng bioengineering Institution, Nanjing, China), respectively, according to the manufacturers’ instructions.

### 5.3. Histology and Morphometry

Renal tissues were fixed in 4% paraformaldehyde and then dehydrated and embedded in paraffin. The paraffin-embedded tissues were sectioned into slices with a thickness of 5 µm and stained with hematoxylin and eosin (HE) [58]. Masson staining of the slides was completed using the Masson Trichrome Stain Kit (Solarbio, Beijing, China)

Transmission electron microscopy was referred to in the previously published literature, and photographs were taken with an HC-1 (80.0 kV, Hitachim, Tokyo, Japan) [59]. Briefly, kidney tissues were fixed with 2.5% glutaraldehyde for 1 day. Then, they were rinsed and cut into 0.5 mm thick strips. After being infiltrated with 2% osmium tetroxide and 1% uranyl acetate, the stained tissues were routinely dehydrated in a methanol gradient and embedded with valerate-12. After polymerization, the ultrastructural features of the liver tissues were observed with transmission electron microscopy and photographed.

### 5.4. Cell Culture

HEK-293 cells were supplied by Nanjing Huaao Biomedical Technology Co., Ltd. (Nanjing, China) and were cultured in Dulbecco’s Modified Eagle’s medium (DMEM) with 1% *v*/*v* penicillin–streptomycin and 10% fetal bovine serum (FBS) in the dark for 30 min at 37 °C in a 5% CO_2_ incubator, and both of these were supplied by the Gibco (Grand Island, NY, USA).

### 5.5. CCK8

Cell viability was measured with the Cell Counting Kit-8 (CCK-8) assay (Beyotime Biotechnology, Shanghai, China) according to the manufacturer’s instructions.

### 5.6. Western Blotting 

The kidney tissues were homogenated and the HEK 293 cells were lysed in RIPA to extract proteins (Beyotime Biotechnology, Shanghai, China). The protein samples were separated via sodium dodecyl sulfate–polyacrylamide gel electrophoresis (SDS-PAGE) and transferred onto PVDF membranes (Merck Millipore Ltd., Tullagreen, Ireland). The membranes were blocked with 5% skimmed milk for 1 h at room temperature and then incubated overnight with primary antibodies (diluted with 1% BSA in TBS–Tween) at 4 °C (Table 1). After washing, the membranes were incubated with an appropriate peroxidase-conjugated secondary antibody (1:1000). The bands were detected with a chemiluminescence system (Bio-Rad, Hercules, CA, USA), and the intensities of the protein bands were measured employing Image J software. 

### 5.7. Statistical Analysis

All the animal experimental data are expressed as means ± standard deviation (SD), and the cell data are presented as means ± standard error (SEM). Student’s *t*-test was used to determine significant differences between the control and MC-LR groups. All statistical analyses were performed using SPSS software version 26.0 (SPSS Inc. Chicago, IL, USA), and *p*  <  0.05 was considered statistically significant.

## Figures and Tables

**Table 1:** Antibodies and sources required for experiments.

Parkin	Abcam, Cambridge, UK	1:2000
p38	Cell Signaling Technology, UK	1:1000
p-p38	Abcam, Cambridge, UK	1:1000
p62	Proteintech, Wuhan, China	1:1000
LC3-II/LC3-I	Proteintech, Wuhan, China	1:500
TOM20	Proteintech, Wuhan, China	1:2000
GAPDH	Proteintech, Wuhan, China	1:50,000

## Data Availability

The datasets used and/or analyzed in the current study are available from the corresponding author upon reasonable request.

## Abbreviations

MC-LR: microcystin-leucine arginine; BUN, blood urea nitrogen; Cr, creatinine; UA, uric acid; ROS, reactive oxygen species; HE, hematoxylin and eosin; SDS-PAGE, sodium dodecyl sulfate–polyacrylamide gel electrophoresis; PVDF, polyvinylidene difluoride; TBST, tris-buffered saline–Tween 20; MKK6, mitogen-activated protein kinase kinase 6; p-p38, phosphorylated p38 mitogen-activated protein kinases; parkin, E3 ubiquitin-protein ligase parkin; p62, sequestosome1; TOM20, mitochondrial outer membrane translocase; LC3-II, microtubule-associated protein light chain 3II; GAPDH, glyceraldehyde-3-phosphate dehydrogenase; CCK8, Cell Counting Kit-8; FBS, fetal bovine serum; DMEM, Dulbecco’s Modified Eagle’s medium.
